# Meat Consumption Associated with the Risk of Chronic Obstructive Pulmonary Disease: A Systematic Review and Meta-Analysis

**DOI:** 10.3390/nu18010006

**Published:** 2025-12-19

**Authors:** Yutong Chen, Hui Xia, Bihuan Hu, Peixuan Tian, Yu Yang, Mi Li, Yajie Zhou, Jing Sui

**Affiliations:** 1 Key Laboratory of Environmental Medicine and Engineering of Ministry of Education, Department of Nutrition and Food Hygiene, School of Public Health, Southeast University, Nanjing 210009, China; 220254251@seu.edu.cn (Y.C.); huixia@seu.edu.cn (H.X.); 220234054@seu.edu.cn (B.H.); 220244286@seu.edu.cn (P.T.); 220244291@seu.edu.cn (Y.Y.); 220254169@seu.edn.cn (M.L.); 2 Jiangsu Engineering Technology Research Center for Modernization of Traditional Chinese Medicine, Nanjing 211102, China; 3 Research Institute for Environment and Health, Nanjing University of Information Science and Technology, Nanjing 210044, China

**Keywords:** meat consumption, chronic obstructive pulmonary disease, a systematic review, meta-analysis

## Abstract

**Background:** Chronic obstructive pulmonary disease (COPD) is a global public health issue and a major cause of morbidity and mortality. Meat consumption is considered one of the factors influencing the risk of COPD. This study aims to perform a systematic review and meta-analysis to synthesize evidence on meat consumption and COPD risk. **Methods:** A systematic review and meta-analysis were performed and reported through a comprehensive search in PubMed and Web of Science from inception to March 2025 (PROSPERO registration ID: CRD42024595137). This meta-analysis included fifteen observational studies. Forest plots were presented, statistical heterogeneity was quantified with the *I*^2^ statistic and investigated through subgroup analyses. Funnel plots and Egger’s test were used to evaluate publication bias. **Results:** The results showed that the odds ratio (OR) for total meat consumption and the risk of COPD was 1.15 (95% confidence interval (CI): 1.01–1.31), suggesting that meat consumption was associated with a higher risk of COPD. Our analysis revealed that fish consumption (OR: 0.84; 95% CI: 0.72–0.97) had a protective effect on COPD risk while processed meat consumption (OR: 1.18; 95% CI: 1.02–1.37) and cured meat consumption (OR: 1.64; 95% CI: 1.41–1.90) was significantly associated with an increased risk of COPD. In addition, subgroup analysis suggested that higher meat consumption was associated with an elevated risk of COPD in cross-sectional study (OR = 1.78; 95% CI: 1.57–2.02), case–control study (OR = 1.52; 95% CI: 1.10–2.10) and in group with 1000 or more participants (OR = 1.16; 95% CI: 1.01–1.33). **Conclusions:** The pooled results of this meta-analysis suggest an association between total meat consumption (encompassing fish, processed meat, cured meat, and unprocessed meat) and COPD. However, the strength of this evidence is tempered by substantial between-study heterogeneity and inconsistent findings across study designs—notably, cohort data failed to support a significant association. Future research should standardize classifications and explore meat subtypes to address heterogeneity.

## 1. Introduction

Chronic obstructive pulmonary disease (COPD) is a chronic and progressive lung disorder characterized by respiratory symptoms and long-term airflow limitation [1]. COPD is the fourth leading cause of death worldwide, posing a significant public health challenge globally [2]. A study has shown that by 2050, the number of people aged 25 and older with COPD globally will increase by 23% compared to 2020. The total number of COPD patients worldwide will approach 600 million, and the number of cases in low-income and middle-income regions will be twice that of high-income regions [3]. Smoking is the primary cause of COPD, additionally, the occurrence of COPD is associated with a variety of other factors, including genetic susceptibility, respiratory infections, and long-term exposure to occupational or environmental harmful substances such as dust, smoke and air pollutants [4].

Epidemiological studies exploring the link between diet and lung function in adults or COPD typically focus on the associations related to the consumption of specific nutrients, foods, or food groups [5], or by focusing on dietary patterns instead of individual nutrients to investigate how diet influences disease [6]. Studies have shown that adopting a prudent or healthy diet pattern like the Mediterranean diet may be a protective factor in preventing and halting the progression of COPD [7]. A study has shown that a traditional dietary pattern (with high intake of meat and potatoes and low intake of soy and grains) is associated with a higher prevalence of COPD [8]. Likewise, a meta-analysis on dietary patterns found that a Western diet high in red and processed meats, unhealthy fats, desserts, and refined grains raises the risk of COPD [9]. Meat consumption seems to be a crucial factor in analyzing the risk factors for COPD. For those who consume meat, it provides basic nutrients such as protein, iron, zinc, vitamin B_12_ and creatine. Various studies have reached different conclusions about the effect of meat consumption on COPD. In this meta-analysis, total meat broadly was defined as a composite category encompassing fish, processed meat, cured meat, and unprocessed meat. This broader classification was necessitated by the heterogeneous reporting of animal protein sources across included primary studies, enabling comprehensive data integration. Though broader than conventional meat definitions, our subsequent subtype analyses clarify the independent effects of each category, addressing the observed divergent subtype-specific outcomes.

Currently, an increasing number of studies have shown that there is a negative correlation between fish consumption and COPD incidence. It is believed that fish has anti-inflammatory effects due to the impact of the *n*-3 polyunsaturated fatty acids eicosatetraenoic acid (EPA) and docosahexaenoic acid (DHA) on arachidonic acid metabolism [10]. An 11-Year Population-Based Cohort Study showed that increasing the intake of fish can reduce the risk of COPD among Chinese women, but not in men [11]. For women, this observational evidence suggested that moderate fish consumption and a comprehensive healthy lifestyle were associated with a lower risk of COPD [11]. A case-control study showed that consumption of cured meat was considerably related to a higher risk of COPD [12]. Additionally, some studies have demonstrated a direct positive association between processed meat consumption and COPD risk. A prospective cohort study involving 289,952 participants found that there is a significant positive correlation between the intake of processed red meat and the risk of COPD [13]. Studies have shown that an unhealthy Western diet is associated with an increased risk of COPD and a faster decline in lung function, notably its high content of processed meats, refined grains, and saturated fats as key contributors to COPD pathogenesis [14]. Among them, the intake of processed meats increases the risk of developing COPD [15]. Currently, there is limited research on unprocessed meat. An investigation showed that there was no significant connection between the consumption of unprocessed meat and an increased risk of COPD [16]. There was no evidence to show that consuming unprocessed meat was related to an increase in COPD incidence [17].

Previous meta-analyses have separately investigated the impact of fish consumption, cured meat consumption, processed meat consumption and unprocessed meat consumption and on COPD. However, evidence specifically focusing on a broad definition of total meat consumption (encompassing fish and other subtypes) remains limited for evaluating its association with COPD. Thus, it is essential to further integrate these findings. Our objective is to conduct a comprehensive systematic review and meta-analysis to collate current findings regarding the correlation between overall meat consumption and COPD incidence.

## 2. Methods

Our study followed the Preferred Reporting Items for Systematic Reviews and Meta-Analyses (PRISMA) in terms of design and reporting [18]. To ensure comprehensive and transparent reporting, the PRISMA 2020 checklists for the full text and abstract are provided in the Supplementary Materials (File S1 and File S2, respectively). This project was registered in the international prospective register of systematic reviews (PROSPERO: CRD42024595137).

### 2.1. Search Methods

Deviations from the registered protocol are as follows: the original registration planned to search four databases (PubMed, Embase, Cochrane Library, and Web of Science), but a preliminary scoping search indicated that the vast majority of relevant observational literature on the relationship between meat consumption and COPD is indexed within PubMed and Web of Science. Therefore, a methodical investigation was performed within the PubMed and Web of Science databases to locate observational research that assesses the relationship between meat consumption and COPD, focusing on articles published online before March 2025. The search terms were as follows: “(Meat OR Processed Meat OR Red Meat OR Cured Meat OR Unprocessed Meat OR Fish OR Fatty Fish OR Diet) AND (Chronic Obstructive Pulmonary Disease OR COPD Mortality OR COPD symptoms (including cough, phlegm, breathlessness, and dyspnea) OR Chronic Bronchitis OR Emphysema)”. This study was limited to English-language publications and included a review of the references in the initial study to ensure comprehensive coverage of related research.

### 2.2. Inclusion and Exclusion Criteria

The criteria for including articles were established as follows: (1) observational epidemiological studies, cohort studies, case-control studies, or cross-sectional studies; (2) the relationship between meat consumption and the risk of COPD; (3) hazard ratio (HR), risk ratio (RR) or odds ratio (OR) and 95% confidence interval (CI); (4) meat consumption (fish, processed meat, cured meat, and unprocessed meat) as an independent variable and COPD risk as a dependent variable. We opted for the newest version for analysis if several datasets were published in separate articles. Articles containing the following content were not cited: (1) correspondence, reviews, and case reports; (2) duplicate studies discovered across various databases; (3) research involving animals or cell studies; (4) studies without necessary risk measurements including HR, RR, or OR.

### 2.3. Data Extraction

The study’s title, data, abstract, and text were examined by two investigators (Y.C. and H.X.) from eligible studies, with any disagreements resolved through discussion, mediation, and consultation with a third researcher (J.S.). The gathered data comprised the publication year, first author’s name, study design type, study duration, region, sample size, gender distribution, adjustments for confounding factors, as well as the OR, HR, or RR associated with meat consumption and COPD risk, along with their corresponding 95% CI. We also extracted detailed exposure information: (a) meat category (fish, processed meat, cured meat, or unprocessed meat); (b) exposure contrast (e.g., highest vs. lowest intake, or per-serving increment).

### 2.4. Quality Assessment

We used the Newcastle-Ottawa Scale (NOS), which is scored from 0 to 9. The scoring parameters are as follows: participants choose up to 4 points, comparability may have up to 2 points, and result evaluation may have up to 3 points [19]. Studies with a NOS score ≥ 7 were classified as high quality. Two reviewers (H.X. and Y.C.) assessed the quality of studies, and discrepancies were addressed through consensus and discussion. Research with particularly low quality may be excluded to ensure that the final result is not overly influenced. To minimize potential biases that may arise from the excessive concentration of specific subgroups or datasets, we conducted sensitivity analyses to assess the impact of each individual dataset on the overall results.

### 2.5. Statistical Analysis

The effect size was represented by OR, RR and HR. All these effect measures were converted to a common logarithmic scale for standardization to ensure comparability across different estimates. Pooling of effect sizes was conducted using the generic inverse-variance method under a random-effects model, which accounts for potential heterogeneity among studies arising from diverse exposure contrasts (e.g., highest vs. lowest meat intake). We used the random effects model and assessed heterogeneity by evaluating Cochran’s Q and *I*^2^. The results were considered to have acceptable heterogeneity when *I*^2^ was less than or equal to 50%. High heterogeneity was indicated when *I*^2^ exceeded 50%. To explore the potential causes of the heterogeneity between studies, we conducted subgroup analyses that included participants’ age range, geographic location, gender, sample size, and study design after identifying significant heterogeneity between studies. For pre-specified subgroup analyses, we rigorously addressed statistical independence of multiple within-study effect estimates via a twofold strategy: estimates from genuinely independent participant subsets (e.g., separate male/female cohorts) were treated as independent data points in relevant subgroup analyses; for those from the same cohort (e.g., associations with fish, red meat, and processed meat in one study), a single, most comprehensive estimate (e.g., “total meat”) was prioritized in the main analysis to preserve independence. Additionally, we conducted sensitivity analyses by excluding individual studies one at a time to evaluate the impact of single studies on the overall pooled results. Begg’s funnel plot and Egger’s test were used to evaluate publication bias, with all statistical analyses conducted using Stata statistical software version 15.0.

## 3. Results

### 3.1. Selected Studies

**Figure 1:**
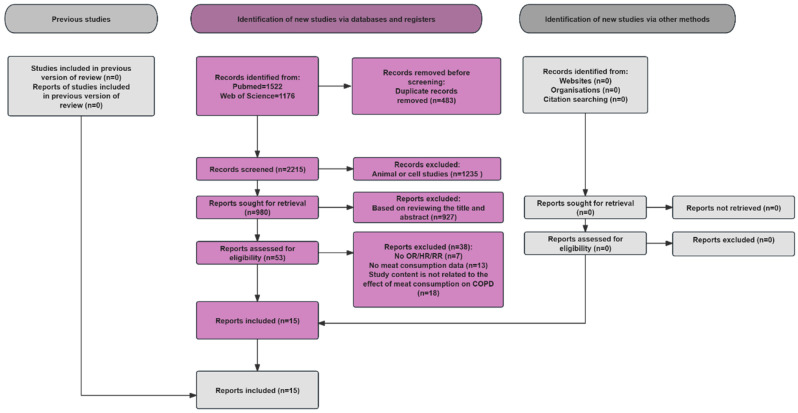
Flow chart of the study selection. COPD, chronic obstructive pulmonary disease; HR, hazard ratio; RR, risk ratio; OR, odds ratio.

The article filtering process is shown in Figure 1. We primarily identified 2698 studies through an article search and screened 2215 after excluding 483 duplicates. After implementing the criteria for inclusion and exclusion, 1235 articles concerning animals and cells were eliminated. Ultimately, we assessed the remaining 53 articles and then identified 38 as unqualified. The reasons were as follows: (1) no data on OR or HR or RR (*n* = 7), (2) no data on meat consumption (*n* = 13), and (3) study content is not related to the effect of meat consumption on COPD (*n* = 18). Ultimately, there were 15 articles in our analysis, comprising 12 cohort studies, 1 cross-sectional study and 2 case-control study.

### 3.2. Characteristics of Studies

Table 1 presents the various features evaluated in this analysis. The total number of participants in 15 articles included in the study is 806,036. Additionally, we categorized the participants based on the types of study designs: there were 12 cohort studies, 1 cross-sectional study, and 2 case-control study. Four studies focused exclusively on males, four on females, while the other seven articles reported overall values and odds ratios (OR) for both male and female groups. Based on geographic distribution, 2 of the 15 researches were carried out in Asian countries, 5 in Europe, and 7 in North America. Participant numbers varied significantly, ranging from 274 to over 252,238. Key adjusted confounders included age, present occupation, body mass index (BMI), educational standard, movement rate, smoking condition, and energy consumption. Based on Newcastle-Ottawa Scale (NOS), the points for the 15 studies ranged from 6 to 9 points, having an average score of 7.8. 13 studies performed above average and were classified as high quality.

### 3.3. Total Meat Consumption and COPD Risk

**Figure 2:**
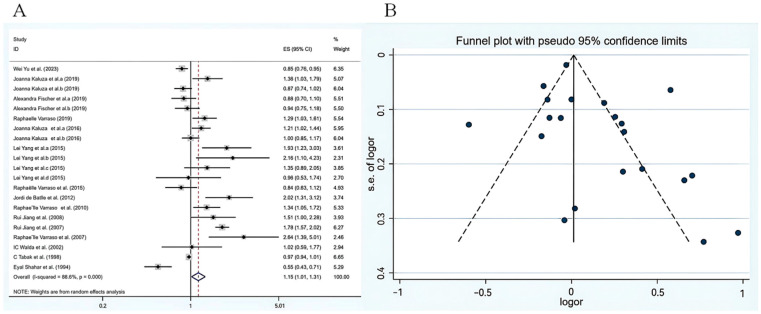
(**A**) Relationship between meat consumption and COPD based on a random effects meta analysis [11,12,16,17,20,21,22,23,24,25,26,27,28,29,30]. (**B**) The funnel plot outcome for the connection between meat consumption and COPD. CI, confidence interval; Black dots: odds ratio value.

A random effects model was adopted to evaluate the association between total meat consumption and COPD risk. The meta-analysis results are illustrated in Figure 2A. The summary estimate of the study indicated that the odds ratio for total meat consumption and COPD risk was 1.15 (95% CI: 1.01–1.31), suggesting that meat intake was associated with a higher risk of COPD. However, considerable heterogeneity was detected among the included studies (*p* < 0.001, *I*^2^ = 88.6%), which substantially limits the robustness and interpretability of this pooled estimate. Therefore, while the point estimate may suggest a potential trend, it should be interpreted with great caution and cannot be regarded as conclusive evidence of an association. To explain the heterogeneity, we conducted analyses considering factors such as geographic location, gender, sample size, and study design. The *p*-value of 0.107 was obtained from the results of the asymmetric Egger linear regression test, while the Begg’s test (Figure 2B) produced a *p*-value of 0.085, suggesting no statistically significant evidence of publication bias. However, it should be noted that the statistical power of these tests may be limited due to the number of included studies, and this result should be interpreted with caution.

### 3.4. Subgroup Analysis of the Effect of Meat Consumption on COPD Risk and Meta Regression

We used meta-regression to identify factors contributing to heterogeneity, assessing the influence of meat type, geographical region, gender, sample size, and study design. The analysis revealed that meat type significantly impacted the effect size (*p* < 0.05). To present the most comprehensive results, we have described the other grouped data, including geographical region, gender, sample size, and study design. The corresponding results are presented in Table 2. Analyzing by meat type revealed a noteworthy negative association between fish consumption and the risk of COPD (OR = 0.84; 95% CI: 0.72–0.97). Processed meat (OR = 1.18; 95% CI: 1.02–1.37) and cured meat consumption (OR = 1.64; 95% CI: 1.41–1.90) were remarkably associated with an increased risk of COPD, whereas no substantial correlation was observed with unprocessed meat consumption (OR = 0.93; 95% CI: 0.81–1.07).

Concerning the regional analysis, we observe no correlation between meat consumption and COPD risk in North American groups (OR = 1.24; 95% CI: 0.87–1.78), European groups (OR = 1.07; 95% CI: 0.92–1.25) and Asian groups (OR = 1.30; 95% CI: 0.87–1.97). Furthermore, subgroup analysis by gender revealed that the effect of meat consumption on COPD risk was not statistically significant (OR = 1.23; 95% CI: 0.91–1.67). There was no significant association between meat consumption and COPD risk for females (OR = 1.08; 95% CI: 0.90–1.30) or males (OR = 1.10; 95% CI: 0.93–1.30). In addition, we categorized studies based on the number of participants, using 1000 people as the cutoff point for grouping. Compared to the group with fewer than 1000 participants (OR = 1.13; 95% CI: 0.77–1.67), the group with 1000 or more participants (OR = 1.16; 95% CI: 1.01–1.33) showed a significantly stronger association with a higher risk of COPD related to meat intake. Eventually, subgroup analysis by study design illustrated that cross-sectional study (OR = 1.78; 95% CI: 1.57–2.02) and case–control study (OR = 1.52; 95% CI: 1.10–2.10) were remarkably associated with a higher risk of COPD. Although cohort studies are considered to provide higher-quality evidence, the pooled result from this subgroup indicated no significant association (OR = 1.05; 95% CI: 0.94–1.16).

Sensitivity analysis revealed that three studies (Jiang, et. al., Shahar, et. al., and Tabak, et. al.) [26,29,30] exerted considerable influence on the meta-analysis. When excluding Jiang et al. [26] alone, heterogeneity was reduced, and the pooled effect size slightly changed to OR = 1.09 (95% CI: 0.98–1.22); When excluding Shahar et al. [29] alone, heterogeneity was reduced, and the pooled effect size changed to OR = 1.19 (95% CI: 1.06–1.35); When excluding Tabak et al. [30] alone, heterogeneity remained reduced, with the pooled effect size being OR = 1.18 (95% CI: 1.01–1.38). This suggested that despite the presence of high heterogeneity, the primary conclusions of the meta-analysis were not significantly altered by the exclusion of this study.

## 4. Discussion

COPD is a leading cause of illness and death worldwide [31]. While numerous factors can impact the risk of COPD, current evidence has brought to crucial importance of nutrition to the risk factor for COPD that can be changed [32]. As a result, the relationship between meat consumption and the risk of COPD raises significant concerns. By combining data from multiple studies, we can achieve more reliable outcomes than relying on individual research. In the meta-analysis, a notable association was observed between total meat consumption and an increased risk of COPD. Specifically, higher intake of processed and cured meat was positively correlated with the occurrence and progression of COPD, whereas fish consumption was associated with a lower risk of COPD, suggesting a potential protective role of fish intake against COPD development.

Our aim was to propose mechanisms that clarify the association between meat consumption and COPD risk. One important mechanism is the protective effect of fish, as its anti-inflammatory properties are crucial for protection against COPD. Fish meat is rich in *n*-3 polyunsaturated fatty acids, mainly eicosapentaenoic acid (EPA) and docosahexaenoic acid (DHA). *N*-6 polyunsaturated fatty acids, such as leukotrienes produced by arachidonic acid metabolism, cause significant bronchoconstriction [33], which increases the airway resistance several times, seriously affects respiratory function, and in severe cases, can lead to complete airway obstruction, causing respiratory failure and other life-threatening conditions. EPA and DHA, on the other hand, as competitive inhibitors of arachidonic acid metabolism, can inhibit the production of pro-inflammatory mediators such as prostaglandins and leukotrienes produced by arachidonic acid as substrates, which helps lower the levels of active inflammatory mediators, such as cyclooxygenase and lipoxygenase systems, thereby exerting a protective effect on the lungs [34,35].

In addition, fish consumption can influence COPD risk by helping to lower the risk of malnutrition. Chronic diseases are generally considered to be positively associated with malnutrition [36,37]. COPD independently increases the risk of malnutrition [38], which in turn increases the risk of COPD or affects disease progression. Fish is rich in high-quality nutrients. First, fish oil can improve muscle circumference in the middle and upper arms [39]; Second, fish, as a common food, provides high-quality protein, and essential amino acids in optimal proportions for human needs. Third, the nutrients found in fish are easily absorbed by the body due to their short muscle fibers, high moisture content, and tender consistency. Furthermore, the low fat and high unsaturated fatty acid content in fish can help reduce cholesterol levels, benefiting cardiovascular and cerebrovascular health [40].

We attempt to further suggest mechanisms by which consuming meat increases the risk of COPD. First, an essential mechanism involves the pro-inflammatory and oxygen-proactive effects of nitrite added to cured and processed meats [16]. Nitrite is added to both processed and cured meats, which acts as a preservative and color fixative agent [41]. Nitrite has pro-oxidative properties that promote reactive nitrogen species production, such as nitrogen dioxide, nitrosyl cations, or nitrous oxide [42,43]. The structural integrity of alveoli relies on intact elastin and collagen, which are critical for maintaining airspace stability. Nitrite (a common additive in processed meats) can induce tyrosine nitration of proteins [34]. These changes stiffen collagen structures, reduce alveolar wall elasticity, and impair lung connective tissue function, contributing to lung tissue damage. Additionally, under inflammatory conditions (e.g., in the presence of myeloperoxidase), nitrite can be converted to highly oxidizing reactive nitrogen species such as peroxynitrite [44,45,46]. These molecules trigger oxidative stress, disrupt lung antioxidant balance, accelerate inflammation, and induce cellular damage (e.g., DNA injury, protein modification, mitochondrial dysfunction) [47]—key pathological processes in COPD.

Another potentially harmful mechanism is related to sodium intake. Processed and cured meat tend to have large amounts of salt added to them. de Batlle et al. suggested that salt entering the body may increase the overall water content in the body and may enhance the negative effects of meat [23]. It is particularly harmful for COPD patients with pulmonary hypertension or poor hemodynamic status, who already have fragile cardiovascular and pulmonary circulatory systems [23].

Finally, another potential mechanism that may increase the risk of COPD is the pro-inflammatory properties that advanced glycation end products (AGEs) possess [48]. Dry heat, ionization, or radiation can significantly accelerate the production of AGEs during food processing [49]. Cooking frequently uses high heat and dehydration, which are meant to make food safer, easier to digest, and more transportable, but they can also contribute to the formation of AGEs [50]. AGEs can promote nutrient overload and accumulation of oxidants, and the ongoing oxidative load may exceed the body’s antioxidant defenses, leading to oxidative stress and chronic inflammation, thereby increasing COPD risk [51].

Our meta-analysis offers some advantages. Firstly, it comprises a substantial sample of 806,036 participants. Unlike previous meta-analyses that focused on a single type of meat, we present a thorough analysis of how all meat consumption affects COPD. Secondly, we sought to propose specific underlying mechanisms to explain the further impact of various meat on COPD risk. We carried out a hierarchical analysis to thoroughly explore how various confounding factors impact the relationship of meat consumption to COPD risk. Our goal is to accurately assess this relationship. Our research represents the first meta-analysis examining the association between total meat consumption and COPD risk. However, this synthesis is preliminary. The overall association was not statistically significant, and the evidence was characterized by substantial heterogeneity across studies. Therefore, the primary value of this work lies in identifying and summarizing the current, limited evidence base, and in highlighting the clear need for more consistent, high-quality prospective research to clarify this potential relationship.

Nonetheless, it is essential to recognize the specific limitations of our study. First, the number of eligible studies examining the association between meat consumption and COPD risk was relatively limited, with only 15 studies included in the final analysis. Second, substantial heterogeneity was observed across the included studies, which may be attributed to variations in study design (e.g., cohort vs. case-control), population characteristics (e.g., age, geographic region, baseline health status), and definitions of meat consumption or COPD outcomes. Third, the small number of studies included in subgroup analyses might introduce bias, limiting the reliability of subtype-specific conclusions. Fourth, as with all observational studies, our analysis cannot establish definitive causal relationships, and residual confounding cannot be fully excluded. Specifically, participants’ dietary patterns are multifaceted (encompassing vegetables, fruits, cereals, and other food groups), and unmeasured or inadequately controlled lifestyle factors (e.g., smoking, alcohol consumption, physical activity) may have contributed to residual confounding or measurement bias. Fifth, our search was restricted to English-language publications, introducing potential language bias that may have excluded relevant non-English literature, further limiting the generalizability of our findings. Finally, the broad definition of total meat, which combined distinct subtypes (e.g., fish, processed meat), may obscure their potentially divergent biological effects. Future research comprising larger-scale, multicenter prospective cohorts with standardized, detailed dietary assessments and capable of exploring these specific subtypes is warranted to clarify these associations.

## 5. Conclusions

The pooled results of this meta-analysis suggest an association between total meat consumption (encompassing fish, processed meat, cured meat, and unprocessed meat) and COPD. However, the robustness of this association is limited by substantial between-study heterogeneity (*I*^2^ = 88.6%, *p* < 0.001) and inconsistent findings across different study designs. Specifically, cross-sectional study (OR = 1.78; 95% CI: 1.57–2.02) and case–control study (OR = 1.52; 95% CI: 1.10–2.10) were remarkably associated with a higher risk of COPD while no significant correlation was found in the analysis of cohort study (OR = 1.05; 95% CI: 0.94–1.16). To explore the source of high heterogeneity and clarify subtype-specific effects, we conducted a subgroup analysis by types of meat, region, gender, sample size, and study design. Notably, this analysis revealed distinct associations for different meat subtypes: fish intake was significantly inversely associated with COPD risk (OR = 0.84, 95% CI: 0.72–0.97), indicating a potential protective effect. In contrast, processed meat (OR = 1.18, 95% CI: 1.02–1.37) and cured meat (OR = 1.64, 95% CI: 1.41–1.90) intake were associated with an increased COPD risk, while unprocessed meat intake showed no significant correlation with COPD risk (OR = 0.93, 95% CI: 0.81–1.07). These findings suggest that the substantial between-study heterogeneity is likely driven by the diverse classification of meat subtypes across included studies, highlighting the need to distinguish between meat types when evaluating their relationship with COPD. Future research should standardize classifications and explore meat subtypes to address heterogeneity.

## Figures and Tables

**Table 1:** General characteristics of the included studies.

Studies	Category	Study Design	Location	Age Range	Sample Size	Gender	Adjustment Variables	NOS
Yu W., et al. [11] (2023)	Fish	cohort study	China	30–79	252,238	All	Body Mass Index (BMI), smoking status, education, fish oil intakes, marry, household income, physical activity, waist circumference, cooking and heating with solid fuel, meat, fresh vegetables and fruit intakes, area of residence, gender	9
Kaluza, J., et al. [16] (2019)	Processed and unprocessed meat	prospective cohort study	Sweden	48–83	34,053	Female	Age, education, BMI, total physical activity, smoking status and pack-years of smoking, alcohol consumption, intake of energy, and recommended food score and modified non-recommended food score	9
Fischer, A., et al. [20] (2019)	Fish and meat	Nested Case-Control Study	Northern Sweden	30–61	370	All	Sex, age, educational level, smoking states, BMI, living alone, total energy	8
Varraso, R., et al. [21] (2019)	Processed meat	cohort study	United States	25–44	87,032	Female	Age, smoking (never, former, current), pack -years of smoking, BMI, physical activity, total caloric intake, US region and race, modified AHEI-2010	8
Kaluza, J. et al. [17] (2016)	Processed and unprocessed meat	prospective cohort study	Sweden	45–79	43,848	Male	Age, educational level, BMI, total physical activity, smoking status and pack-years of smoking, intake of energy, alcohol consumption, recommended food score and non-recommended food score	7
Yang, L. et al. [12] (2015)	Cured meat	case-control study	China	NA	3188	All	Pre-existing tuberculosis, smoking, passive smoking, occupational exposure to metallic toxicant, poor housing ventilation, biomass burning, cured meat consumption, and seldom vegetables/fruits consumption	6
Varraso, R., et al. [22] (2015)	Fish	cohort study	United States	30–75	120,175	All	Age, smoking status, pack-years of smoking, pack-years squared of smoking, secondhand tobacco exposure, race-ethnicity, physician visit, US region, spouse`s highest educational attainment, menopausal status, BMI, physical activity, multivitamin use, energy intake, and modified prudent and Western dietary patterns	8
de Batlle, J., et al. [23] (2012)	Cured Meat	cohort study	Spain	60–76	274	All	Age, FEV_1_, and total caloric intake	7
Varraso, R., et al. [24] (2010)	Cured Meat	prospective cohort study	United States	40–75	111,580	All	Age, sex, smoking, energy intake, BMI, US region, physician visits, physical activity, diabetes, and intakes of omega-3 and cured meat	9
Jiang, R., et al. [25] (2008)	Cured Meat	prospective cohort study	United States	38–63	71,531	Female	Age, smoking, and multiple other potential confounders	9
Jiang, R., et al. [26] (2007)	Cured Meat	Cross-sectional study	United States	≥45	7352	All	Age, smoking, and multiple other potential confounders	6
Varraso, R., et al. [27] (2007)	Cured Meat	prospective cohort study	United States	40–75	42,915	Male	Age, smoking status, pack-years, pack-years squared, energy intake, race/ethnicity, US region, BMI, and physical activity	9
Walda, I.C., et al. [28] (2002)	Fish	prospective cohort study	Europe	50–69	2917	Male	Country, age and smoking	8
Tabak, C., et al. [29] (1998)	Fish	Cohort study	United States, Italy, ex-Yugoslavia, The Netherlands, Finland, Japan, Greece	50–69	12,763	Male	Age, total energy intake, prevalence of cigarette smoking, work-related activity level and BMI	7
Shahar, E., et al. [30] (1994)	Fish	prospective cohort study	United States	45–64	15,800	All	Pack-years of smoking, age, sex, race, height, weight, energy intake, and educational level	7

NOS, Newcastle-Ottawa scale.; FEV_1_, forced expiratory volume in 1 s; AHEI-2010, Alternate Healthy Eating Index 2010.

**Table 2:** Subgroup analysis of meat and the risk of COPD.

Subgroup	No. of Study	OR (95%CI)	*I* ^2^
Types of meat			
Fish	6	0.84 (0.72, 0.97)	79.5%
Processed meat	4	1.18 (1.02, 1.37)	46.2%
Cured meat	6	1.64 (1.41, 1.90)	34.7%
Unprocessed meat	2	0.93 (0.81, 1.07)	31.2%
Region			
America	7	1.24 (0.87, 1.78)	92.7%
Europe	5	1.07 (0.92, 1.25)	70.0%
Asia	2	1.30 (0.87, 1.97)	81.5%
Gender			
Male	4	1.10 (0.93, 1.30)	73.9%
Female	4	1.08 (0.90, 1.30)	72.0%
All	7	1.23 (0.91, 1.67)	93.0%
Sample size			
<1000	2	1.13 (0.77, 1.67)	82.8%
>=1000	13	1.16 (1.01, 1.33)	89.7%
Study design			
Cohort study	12	1.05 (0.94, 1.16)	80.9%
Cross-sectional study	1	1.78 (1.57, 2.02)	NA
Case-control study	2	1.52 (1.10, 2.10)	36.6%

## Data Availability

No new data were created or analyzed in this study.

## Supplementary Materials

The following supporting information can be downloaded at: https://www.mdpi.com/article/10.3390/nu18010006/s1. File S1: PRISMA 2020 checklist; File S2: PRISMA 2020 abstract checklist.
